# Autonomic and vascular function testing in collegiate athletes following SARS-CoV-2 infection: an exploratory study

**DOI:** 10.3389/fphys.2023.1225814

**Published:** 2023-07-17

**Authors:** J. Carter Luck, Cheryl Blaha, Aimee Cauffman, Zhaohui Gao, Amy C. Arnold, Jian Cui, Lawrence I. Sinoway

**Affiliations:** ^1^ Milton S. Hershey Medical Center, Penn State Heart and Vascular Institute, Pennsylvania State University College of Medicine, Hershey, PA, United States; ^2^ Department of Neural and Behavioral Sciences, Pennsylvania State University College of Medicine, Hershey, PA, United States

**Keywords:** SARS-CoV-2, COVID-19, autonomic function, athletes, vascular function, blood pressure

## Abstract

**Introduction:** Recent studies suggest that SARS-CoV-2 infection alters autonomic and vascular function in young, otherwise healthy, adults. However, whether these alterations exist in young competitive athletes remains unknown. This study aimed to assess the effects of COVID-19 on cardiac autonomic control and vascular function in collegiate athletes who tested positive for COVID-19, acknowledging the limitations imposed by the early stages of the pandemic.

**Methods:** Sixteen collegiate athletes from various sports underwent a battery of commonly used autonomic and vascular function tests (23 ± 9, range: 12–44 days post-infection). Additionally, data from 26 healthy control participants were included.

**Results:** In response to the Valsalva maneuver, nine athletes had a reduced early phase II blood pressure response and/or reduced Valsalva ratio. A depressed respiratory sinus arrhythmia amplitude was observed in three athletes. Three athletes became presyncopal during standing and did not complete the 10-min orthostatic challenge. Brachial artery flow-mediated dilation, when allometrically scaled to account for differences in baseline diameter, was not different between athletes and controls (10.0% ± 3.5% vs. 7.1% ± 2.4%, *p* = 0.058). Additionally, no differences were observed between groups when FMD responses were normalized by shear rate (athletes: 0.055% ± 0.026%/s-1, controls: 0.068% ± 0.049%/s-1, *p* = 0.40).

**Discussion:** Few atypical and borderline responses to autonomic function tests were observed in athletes following an acute SARS-CoV-2 infection. The most meaningful autonomic abnormality being the failure of three athletes to complete a 10-min orthostatic challenge. These findings suggest that some athletes may develop mild alterations in autonomic function in the weeks after developing COVID-19, while vascular function is not significantly impaired.

## 1 Introduction

The highly infectious severe acute respiratory syndrome coronavirus 2 (SARS-CoV-2), which causes coronavirus disease 2019 (COVID-19), can cause a range of symptoms and complications, affecting various systems in the human body. Recent studies have identified concerning impairments in autonomic and vascular function in young otherwise healthy individuals who are recovering from COVID-19 (Nandadeva et al., 2021; Ratchford et al., 2021; Stute et al., 2021). These impairments are particularly ominous findings in young adults, as it is well-established that both autonomic and vascular endothelial dysfunction play a significant role in the development of cardiovascular disease (Bonetti et al., 2003; Thayer et al., 2010). Physical activity is important for overall health and wellbeing, and it is especially important during the COVID-19 pandemic. Whether similarly aged competitive athletes, who are generally regarded as a physically fit and healthy individuals, develop similar dysautonomia and vascular dysfunction secondary to a SARS-CoV-2 infection remains unclear.

Initial uncertainty regarding the cardiovascular outcomes following a SARS-CoV-2 infection (Puntmann et al., 2020) prompted a rapid response by the Big Ten Conference early in the pandemic, which required each of its 14 institutions to screen all student-athletes that tested positive for COVID-19 using a comprehensive cardiac evaluation, before returning to competition. The Big Ten Conference later published its registry data of 1,597 athletes, which identified that 2.3% of athletes infected with SARS-CoV-2 developed clinical and subclinical myocarditis (Daniels et al., 2021). Following these initial (Nandadeva et al., 2021; Ratchford et al., 2021; Stute et al., 2021) and follow-up studies (Stute et al., 2022), the question of whether any alterations in autonomic or vascular function exist in collegiate athletes following SARS-CoV-2 infection remains unanswered and is the purpose for the present investigation.

Identifying alterations in autonomic function in athletes with an acute SARS-CoV-2 infection may be particularly interesting for several reasons. Importantly, it is recognized that individuals with vagal dysfunction may experience a reduction in exercise capacity(Machhada et al., 2017). Moreover, it is a topic of ongoing debate whether regular physical activity could have a protective impact against severe COVID-19 outcomes (da Silveira et al., 2021; Steenkamp et al., 2022). Indeed, the cardio-protective benefits of regular exercise have been meticulously documented for years (Blair, 1995; Joyner and Green, 2009), and evidence suggests that regular physical activity could also benefit the immune system (Nieman and Wentz, 2019). Therefore, the possibility that regular exercise could lead to less severe outcomes in athletes, who presumably engaged in consistent physical activity before a SARS-CoV-2 infection, is particularly intriguing. Moreover, understanding the autonomic control of heart rate (HR) and blood pressure (BP) in young athletes can be accomplished via a number of safe, standardized non-invasive interventions (Novak, 2011). Thus, this investigation aimed to document any acute alterations in autonomic or vascular function in National Collegiate Athletic Association Division I athletes who had tested positive for SARS-CoV-2 in the prior weeks. To uncover any potential alterations in autonomic or vascular function, a battery of commonly used autonomic function tests were selected using previously described methods (Novak, 2011). However, an acknowledgement to the limitations posed by the COVID-19 safety regulations in designing the ideal research plan that made it difficult to obtain direct comparisons with a COVID-negative athlete control group, the present study is intended to be an exploratory first step in this unique population.

## 2 Methods

### 2.1 Participants and study design

This study followed a cross-sectional design and was approved by the Penn State Hershey Institutional Review Board (IRB# 16298) and conformed with the Declaration of Helsinki. All Penn State athletes who tested positive for SARS-CoV-2 and were undergoing clinical clearances to return to sports were recruited through the Penn State Athletic Department and Sports Medicine providers. Prior to enrollment, all athletes were seen by a healthcare provider, where a surface 12-lead electrocardiogram, and limited echocardiography study was performed to evaluate cardiac function and structure. All athletes were first educated on the study purpose and protocols before providing written informed consent to participate. All protocols were performed at the Penn State Heart and Vascular Institute within the Clinical Research Center in the Clinical and Translational Science Institute, located on the Penn State Hershey campus.

Sixteen collegiate athletes (12 men, 4 women, 20 ± 1 years, 181 ± 10 cm, 81 ± 18 kg, 24.6 ± 3.1 kg/m2), who tested positive for SARS-CoV-2 using a nasopharyngeal swab polymerase chain reaction (PCR) assay, were studied between November 2020 and February 2021. *A priori* power calculation to determine the required sample size for our study initially aimed to include a sample size of 25 athletes who had COVID-19. Given that we were only able to obtain a sample size of 16 athletes for our study, it is important to acknowledge that the statistical power of our analysis may be limited. Athletes consisted of competitive lacrosse players (*n* = 7 men), ice hockey players (*n* = 2 women), a fencer (*n* = 1 man), a football player (*n* = 1 man), a golfer (*n* = 1 man), a gymnast (*n* = 1 man), a soccer player (*n* = 1 woman), a swimmer (*n* = 1 man), and a tennis player (*n* = 1 woman). Two additional groups, consisting of 26 young adults who did not test positive for SARS-CoV-2, served as control participants: one group (*n* = 10, 7 men, 3 women) for the brachial artery flow-mediated dilation (FMD) test, and a group (*n* = 16, 4 men, 12 women) for the orthostatic challenge (Table 1). Out of the 16 athletes who had COVID-19, 14 were identified as white, one as Asian, and one as more than one race. In terms of ethnicity, 15 participants were classified as non-Hispanic, and onw participant identified as Hispanic. Among the 17 participants in the orthostatic control group, 12 were identified as white, 3 as Asian, 1 as more than one race, and 1 as Black/African American. In terms of ethnicity, 13 participants were classified as non-Hispanic, and 3 participants identified as Hispanic. All participants in the FMD control group were identified as white non-Hispanic. The menstrual cycle phase in women participants was documented. Among the 4 women in the COVID group, one woman was on day 4 of her menstrual cycle, while the other three women reported having irregular menstrual history, with one of them using an IUD as a contraceptive method. In the orthostatic control group, consisting of 12 women, participants reported being on various days of their menstrual cycle: days 1, 10, 10, 11, 15, 15, 21, 24, and 25. Additionally, three participants in the orthostatic control group reported having irregular menstrual history. No menstrual cycle data was collected in the three women in the FMD control group.

**TABLE 1:** Subject characteristics. The total number of symptoms are a count of 10 symptoms (shortness of breath or difficulty breathing, fatigue, new loss of taste or smell, nausea vomiting or diarrhea, fever or chills, cough, muscle or body aches, headache, sore throat, and/or congestion or runny nose) where 0 represents and asymptomatic individual. The symptom score is presented as an eighteen-point scale where 0 is asymptomatic and 18 is the most severe symptom score. BMI; body mass index, PCR; polymerase chain reaction, SpO2; oxygen saturation, FMD; flow-mediated dilation. HRV; heart rate variability, SDNN; mean standard deviation of the normal sinus beats, RMSSD; root mean square of successive differences between normal heartbeats, LF; low frequency, HF; high frequency. Values are mean ± SD unless otherwise specified. Two-tailed Student's t test were used to compare outcomes between each control group (*n* = 16) and (*n* = 10) and SARS-CoV-2 positive athletes (*n* = 16). *p < 0.05, between groups.

	SARS-Cov-2-positive (athletes)	SARS-Cov-2-negative (orthostatic control)	SARS-Cov-2-negative (FMD control)		
	*n* = 16 (12 men/4 women)	*n* = 16 (4 men/12 women)	*n* = 10 (7 men/3 women)	*p*	*p*
Age, years	20 ± 1	28 ± 6*	22 ± 2*	< 0.01	< 0.01
Height, cm	180.5 ± 10	166.9 ± 6.9*	176.5 ± 11	< 0.01	0.36
Weight, kg	81.1 ± 18	63.5 ± 10.1*	76 ± 13.9	< 0.01	0.45
BMI, kg/m^2^	24.6 ± 3.1	22.8 ± 3.2	24.2 ± 2	0.11	0.70
Oral contraceptive use, % women	50%	42%	—	0.86	—
Supine systolic arterial pressure, mm Hg	111 ± 7	101 ± 6*	117 ± 9	< 0.01	0.09
Supine diastolic arterial pressure, mm Hg	63 ± 7	62 ± 8	72 ± 8*	0.55	0.01
Supine mean arterial pressure, mm Hg	79 ± 6	76 ± 6	87 ± 7*	0.18	< 0.01
Heart rate, bpm	59 ± 10	64 ± 7	74 ± 14*	0.11	< 0.01
HRV measures	—	—	—	—	—
SDNN, ms	81 ± 31	66 ± 29	—	0.14	—
RMSSD, ms	78 ± 47	63 ± 41	—	0.34	—
Total power, ms^2^	9647 ± 8249	5027 ± 4905	—	0.06	—
LF power, ms^2^	2402 ± 1945	1073 ± 904*	—	0.02	—
LF power, n.u	34 ± 16	26 ± 10	—	0.11	—
HF power, ms^2^	3575 ± 4035	2444 ± 3511	—	0.40	—
HF power, n.u	37 ± 19	45 ± 16	—	0.18	—
LF/HF ratio, %	1.48 ± 1.59	0.71 ± 0.4	—	0.06	—
Respiratory rate, cyc/min (M[range])	15 [23–10]	—	—	—	—
SpO2 (%)	98 ± 1	—	—	—	—
Time since positive PCR assay, days	23 ± 9	—	—	—	—
Total number of symptoms (0–10)	2 ± 2	—	—	—	—
Length of symptoms, days	4 ± 6	—	—	—	—
Symptom score, (0–18)	4 ± 3	—	—	—	—

The control participants in the FMD experiments were studied prior to the first confirmed case of COVID-19 in the United States, on 19 January 2020, while the control participants in the orthostatic challenge were studied after February 2022. The participants studied after the start of the pandemic did not have a COVID-19 diagnosis at any time. All student-athletes participating in our study were required to adhere to quarantine guidelines, which prohibited them from practicing or competing for a 14-day period, or until obtaining two consecutive negative PCR tests. They were retested on a weekly basis until they received a negative test result. Apart from the SARS-CoV-2 infection in the athletes, all participants were apparently healthy and without any known cardiac, metabolic, or renal diseases. Participants were not taking any medications that target the cardiovascular system (e.g., beta-blockers, nitrates, sympathetic adrenergic agonists or antagonists, cholinergic agonists or antagonists, calcium channel blockers, diuretics, statins, or ACE inhibitors). All athletes and control participants abstained from caffeine for at least 8 hours and from alcohol and over-the-counter medications for at least 12 h prior to the experimental visit.

### 2.2 Instrumentation and data collection

Athletes were asked to complete an in-house COVID-19 symptom severity survey as well as a health history questionnaire on the day of testing. Athletes were instructed to self-select “yes” or “no” using a list of symptoms, which consisted of shortness of breath or difficulty breathing, fatigue, new loss of taste or smell, nausea or vomiting or diarrhea, fever or chills, cough, muscle or body aches, headache, sore throat, and/or congestion or runny nose. Symptoms of shortness of breath or difficulty breathing, fatigue, new loss of taste or smell, nausea or vomiting or diarrhea were assigned 3 points, as these symptoms may be indicative of more severe infection. All other symptoms, which are also symptoms of the common cold, were assigned 1 point. Weighted symptoms were totaled and presented on an eighteen-point scale, where a minimum score of 0 was considered asymptomatic and a maximum score of 18 was considered the most severe symptom score.

The primary variables of interest included BP, HR, recovery time of HR from stress, HR variability (HRV). Additionally, brachial artery FMD and reactive hyperemia were assessed using a Doppler ultrasound system (GE Logiq eR7 and L4-12T-RS transducer, GE Medical Systems, Milwaukee, WI). An antecubital intravenous catheter was placed, and a blood sample was obtained and sent to the Penn State Clinical Laboratory for measurement of renin activity, aldosterone, c-reactive protein, cardiac troponin, and a complete blood count (CBC). Brachial artery systolic BP (SBP), diastolic BP (DBP) were monitored with an automatic noninvasive sphygmomanometer (Philips Sure Signs VS3), continuous beat-by-beat BP was recorded using a finger cuff and the volume-clamp method (Finometer, FMS, Netherlands). Beat-by-beat mean arterial pressure (MAP) was calculated as 1/3 SBP + 2/3 DBP in LabChart version 7 software. A three-lead EKG (Cardiocap/5, GE Healthcare) was used to record HR. A pneumography belt was placed around the abdomen to measure respiratory movement.

*Autonomic Function Testing*. All athletes underwent a battery of standardized autonomic function tests: 1.) 10-min resting baseline, 2.) Valsalva maneuver (VM), 3.) respiratory sinus arrhythmia (RSA) test, consisting of a paced respiratory frequency of six breaths per minute lasting 1 minute, 4.) fatiguing isometric handgrip exercise at 30% of maximal voluntary contraction, and 5.) an orthostatic challenge of standing for a maximum of 10 minutes. All protocols were carried out in in a quiet, dimly lit, thermoneutral (22°C) laboratory. The intervals between tests were > 5 min or until hemodynamic variables stabilized.

After an acclimation period (in a supine position for at least 5 min or until hemodynamic variables stabilized), baseline measurements were conducted, consisting of 10 min of uninterrupted rest. A blood sample was taken at the completion of this resting baseline. Next, athletes were instructed to perform the VM while forcibly exhaling into a mouthpiece for 15 s while maintaining an expiratory pressure of 40 mm Hg. A prerecorded sound cue was used to standardize the strain time. The expiratory pressure could be viewed by each athlete via digital display. After a practice attempt, each athlete performed no more than three successive VM and each maneuver was separated by no less than 60 s. If the subject was unable to maintain a pressure >30 mm Hg for a minimum of 10 s, the data from the maneuver was not included in the analysis.

The RSA test was used to assess parts of the parasympathetic nervous system, including the vagus nerve, that are involved in the regulation of HR (Novak, 2011). Athletes were instructed to take deep breaths which were paced to an oscillating tone (6 breaths/minute for 1 minute). The RSA was determined by identifying the maximum and minimum HR during each inspiration/expiration cycle and calculating the average difference across the cycles.

To assess the exercise pressor response, athletes performed isometric handgrip exercise at 30% of their maximal voluntary contraction until fatigue. Before beginning a pre-exercise baseline, athletes performed three successive maximal voluntary isometric handgrip contractions, each separated by 60 s. After determining peak maximal voluntary contraction, athletes rested for 5 minutes, followed by a baseline data collection period of 1 minute. The 30% of maximum was calculated and set on a visual device so each athletes gripped appropriately.

Athletes next underwent an orthostatic challenge to assess the autonomic response to postural change. Starting from a resting supine position, athletes transitioned to an upright standing position for a maximum of 10 min or until presyncope symptoms were observed. Participants were instructed to quietly stand with both arms supported at heart level to assess finger and brachial BP. A research nurse monitored patients for the development of any presyncope symptoms. Presyncope symptoms were defined as a sustained SBP of < 80 mm Hg, or periodic SBP of <80 mm Hg associated with symptoms of lightheadedness, nausea, diaphoresis, and/or bradycardia. Similarly, in a group of healthy but non-competitive control participants (*n* = 16), hemodynamic variables were recorded for 10 min at supine position under resting condition. Thereafter, the participants transitioned to an upright standing position for a maximum of 10 min or until presyncope symptoms were observed.

*Brachial artery flow-mediated dilation and reactive hyperemia*. Brachial artery FMD was assessed in 14 of the 16 collegiate athletes (10 men, 4 women, 20 ± 1 years, 179 ± 9 cm, 76.6 ± 10.8 kg, 23.8 ± 1.6 kg/m2) and ten non-competitive controls (7 men, 3 women, 22 ± 2 years, 176 ± 11 cm, 76.0 ± 13.9 kg, 24.2 ± 2.0 kg/m2). All FMD protocols were conducted by an experienced registered diagnostic cardiac sonographer (ZG) while adhering to previously established methodology (Harris et al., 2010). Participants were assessed in the supine position with their arm extended ∼80°. A pneumatic cuff placed around the proximal forearm and connected to compressed gas with a ball valve for allowing for rapid inflation/deflation. Prior to inflating the pressure cuff, baseline measurements were obtained following 10 min of uninterrupted rest. Three to five baseline diameter measurements were captured at end-diastole. The pressure cuff was next rapidly inflated to 200 mm Hg and maintained for 5 minutes, during which full arterial occlusion was verified by continuous Doppler ultrasound imaging. The cuff was deflated, and post-occlusion measurements of brachial artery diameter and velocity were obtained. Specifically, the peak hyperemic velocity during the first 20 s was captured using Doppler ultrasound, followed by an immediate switching to 2D imaging to capture brachial artery diameter. Brachial artery diameter and velocity were recorded for 3 minutes and analyzed offline by an experienced blinded investigator (KB) using commercially available software (ProSolv^®^ CardioVascular analyzel 3.0, Indianapolis, Indiana). To account for any differences in baseline diameter, an allometric scaling analysis was performed (Atkinson and Batterham, 2013). Additionally, shear rate was calculated using the following equation: Shear rate (s−1) = 8·Vmean (cm/s)/internal vessel diameter (cm). To normalize the vasodilation for shear rate, FMD was divided by the cumulative shear rate (%Δdiameter/s−1 s) (Pyke and Tschakovsky, 2005).

The order for the autonomic function test and FMD test in collegiate athletes was randomized. These two sets of the tests were performed in separate laboratories and the interval between the tests were > 90 min. The autonomic function tests were not performed in the control participants who underwent the FMD test.

### 2.3 Data analysis

All signals were continuously recorded at 1,000 Hz via a multi-channel analog-to-digital data acquisition system (PowerLab, AD Instruments, Colorado Springs, CO) and analyzed in LabChart version 7 software. Five-minute recordings of HRV were calculated via linear algorithms and analyzed in the time domain and frequency domain, in accordance with the precious recommendations for short-term recordings, using LabChart version 8 software (LabChart 8, AD Instruments, Colorado Springs, CO). The averaged values of all HRV indices, from two sequential 5-min recordings during the 10-min baseline were reported. All ectopic beats were excluded from the analysis. Low (LF: 0.04–0.15 Hz) and high-frequency (HF: 0.15–0.4 Hz) data are expressed in absolute (ms2) and normalized (nu) units and the LF/HF ratio was calculated (Task Force of the European Society of Cardiology the North American Society of Pacing Electrophysiology, 1996). The beat-by-beat values of HR and BP were exported into a data file, the baroreflex analysis was conducted via the sequence method (3-beat minimum detection) using the opensource software CardioSeries by (Dias, D.M.P, www.danielpenteado.com).

**FIGURE 1:**
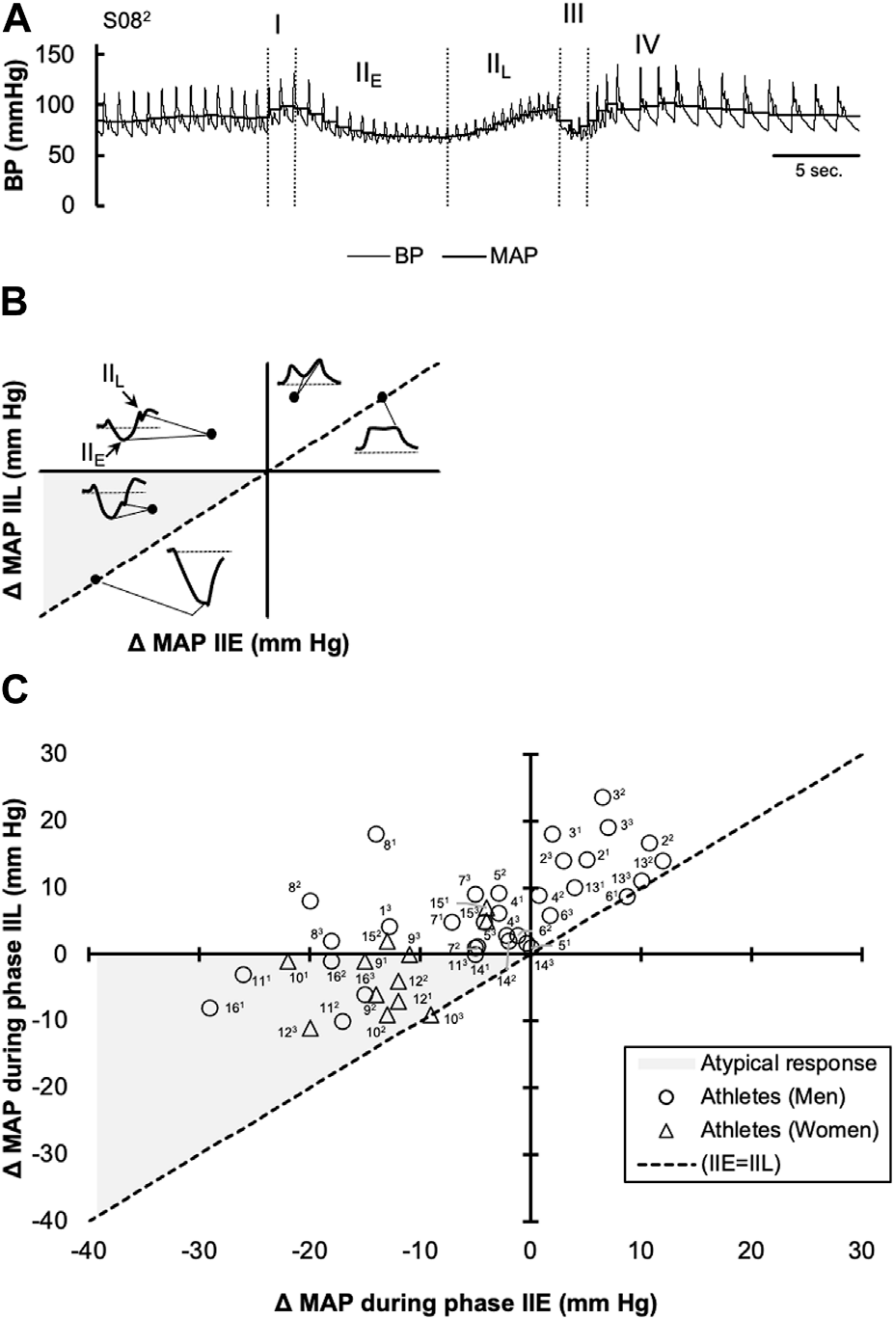
Changes in mean arterial pressure (MAP) in response to the Valsalva maneuver (VM) in collegiate athletes with COVID-19. A representative continuous blood pressure (BP) and calculated mean arterial BP (MAP) tracing with phases I, II (i.e., IIE and IIL), III, and IV is shown in **(A)** A diagram illustrating the expected location for several various MAP patterns that can be observed during the VM is shown via a coordinate system in **(B)**. Individual values, obtained during phases IIE (*x*-axis) and IIL (*y*-axis) during each of the three sequential VM trials are shown using a coordinate system **(C)**. The line *y* = *x* (dotted) represents the points where IIE and IIL converge (i.e., where phase IIL is absent). Individual responses that are located in quadrant II were considered typical findings, while those located along the line *y* = *x*, within quadrant I, were considered to follow a “square-wave” pattern. Individual responses falling below the *x*-axis in the shaded grey within quadrant III, indicate a failure in MAP recovery during IIL, were considered atypical (Low, 1993; Novak, 2011).

For data collected during the VM, baseline averages of BP and HR were determined from a 30-s period immediately preceding each VM. Consistent with previous reports (Sandroni et al., 1991; Davis and Crandall, 2010; Stavres et al., 2023), BP and HR responses during each VM were divided into four distinct phases (I, II, III, and IV) based off the beat-by-beat MAP, with phase II subdivided into early (IIE) and late (IIL), respectively (Figure 1A.). Phase IIE and IIL were differentiated by the nadir in MAP during the strain period. MAP at the end of phase IIE and end of phase IIL were used in the analysis. The autonomic responses to each VM maneuver were quantified by the absolute nadirs (maximum and minimum values) recorded for MAP during each phase. The Valsalva ratio was determined by the maximum HR during the strain period divided by the minimum HR obtained within the 30 s following the peak HR. Once data were collected, MAP and cardiac rhythm were analyzed offline (LabChart 8 Pro, AD Instruments, Colorado Springs, CO) and averaged across each individual cardiac cycle. SBP and DBP were identified as the maximum and minimum values within each cardiac cycle, respectively, and used to calculate a beat-by-beat pulse pressure (PP) and MAP.

### 2.4 Statistical analysis

Between-group comparisons of subject characteristics were made using a Student’s *t*-test and Shapiro-Wilk test did not show a significant departure from normality. Pearson’s correlation coefficient was calculated to assess any correlations between symptom scores and various indices of autonomic function. All statistical analyses were performed using SPSS statistical analysis software (SPSS Statistics version 28, IBM Corp., Aramonk, NY), and all data are presented as mean ± standard deviation (SD), or as individual raw values and significance was accepted at *p* ≤ 0.05.

## 3 Results

### 3.1 Baseline subject characteristics

The subject characteristics are presented in Table 1. As indicated in this table, the 16 athletes were studied 23 ± 9 (range: 12–44) days after testing positive for SARS-CoV-2. One athlete was tachypneic with a pulse oxygen saturation of 98%. All other athlete’s vital signs were normal (Table 1), and echocardiography showed normal heart structure and function with resting ejection fractions of 62% ± 5%. As indicated in Table 1, comparisons of indices of HRV during the 10-min baseline, were significantly different between athletes and 16 control participants in only low frequency (LF) power (2,401.6 ± 1,944.6 vs 1,073.1 ± 904.2 ms2, *p* = 0.02). All other indices of HRV showed no significant differences between athletes and control participants (Table 1). When compared to a group of ten aerobically trained athletes (Aubert et al., 2001), athletes with COVID-19 showed no differences in resting SDNN (81 ± 31 vs. 97.9 ± 15.7 ms, *p* = 0.051), RMSSD (78 ± 47 vs. 73.5 ± 23.7 ms, *p* = 0.72), and pNN50 (40.7% ± 27.4% vs. 40.1% ± 16.6%, *p* = 0.94). Additionally, resting cardiac baroreflex sensitivity was not different between the athletes and the 16 orthostatic healthy control group (28.9 ± 15.4 vs. 19.9 ± 8.9 msec/mmHg, *p* = 0.06).

Analysis of blood biomarkers showed that all athletes were negative for cardiac troponin (all values <0.010 ng/ml). Four athletes had elevated plasma renin activity (between 1.9–4.8 ng/mL/hr) and of these four individuals, one also had elevated plasma aldosterone levels (25.8 ng/dL). Additionally, two female athletes had low red blood cell count (4.14 and 4.31 M/uL), hemoglobin (12.3 and 12.9 g/dL), and hematocrit (38.4% and 37.9%) which was attributed to their menstrual cycle. One male athlete had an elevated mean corpuscular volume (96.1 fL), while the group mean was within normal limits (89 ± 3 fL). One male athlete had depressed red cell distribution width (11.3%), while the group mean was within normal limits (12.3% ± 0.8%). Otherwise, levels of C-reactive protein (0.08 ± 0.07 mg/dL), white blood cells (5.7 ± 1.4 K/uL), mean corpuscular hemoglobin (30 ± 1 pg), mean corpuscular hemoglobin concentration (33 ± 1 g/dL), platelet count (237 ± 38 K/uL), and mean platelet volume (10.0 ± 0.8 fL) were within normal limits in all athletes.

### 3.2 Responses to Valsalva maneuver

Participants maintained expiratory pressures of 31 ± 9, 39 ± 6, and 39 ± 5 mm Hg during phases I, IIE, and IIL, respectively. However, one athlete did not maintain consistent expiratory pressure during their first and second VM attempts. Thus, these data were excluded from the analysis. MAP responses during phases IIE and IIL are plotted in Figure 1. As indicated in this figure, values are change from baseline (i.e., the mean MAP during the 30 s prior to each VM) and the dashed line (*y* = *x*) represents an absence in a MAP inflection point were MAP values during phases IIE and IIL converge. In each of the three VMs, 10 athletes had a reduction in MAP during phase IIE (−11 ± 8 mm Hg). Conversely, in two athletes, the phase IIE inflection point occurred above the baseline (7 ± 4 mm Hg) during each of the three successive VMs. Lastly, two athletes showed inconsistent phase IIE responses across each of the three VMs (inflection points ranging from −3–9 mm Hg). During phase IIL, MAP returned or surpassed baseline in 11 athletes (8 ± 6 mm Hg), while in five athletes, phase IIL MAP values remained below the baseline. Pulse pressure was reduced during phase II (i.e., IIE + IIL) by 25% ± 22%. During phase II, a “square wave” pattern was observed in four athletes in at least two of the three VMs. During phase IV, MAP surpassed the baseline by 11 ± 8 mm Hg. The average maximum HR during the VM was 88 ± 18 bpm and the minimum HR, obtained within 30 s of the peak HR, was 52 ± 10 bpm. The average Valsalva ratio was 1.77 ± 0.63.

### 3.3 Responses to slow breathing

**FIGURE 2:**
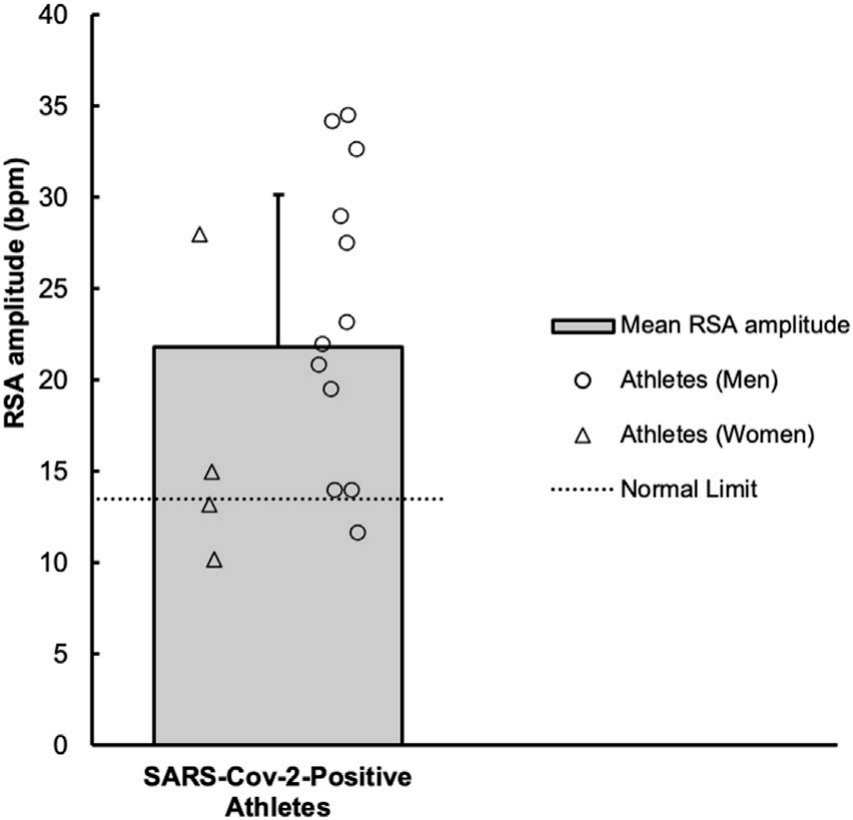
Values of average respiratory sinus arrhythmia (RSA) amplitude, across the six respiratory cycles, in collegiate athletes with COVID-19 are shown. A horizontal dotted line represents a previously established “normal” threshold, for individuals aged 10–29 years (Novak, 2011). Values below 14 beats/minute (*n* = 3) were considered atypical.

The RSA amplitude values are shown in Figure 2. In athletes, the average baseline HR prior to paced breathing was 60 ± 8 bpm. Across the six inspiratory breaths, the average HR increased above baseline to 74 ± 10 bpm. Subsequently, the average HR across the six expiratory breathes decreased to 52 ± 8 bpm. The RSA amplitude was 22 ± 8 bpm. When compared to one previous investigation (Ewing et al., 1985) of 71 healthy participants ranging in ages from 16 to 65, the mean RSA amplitude was reduced in athletes with COVID-19 (31 ± 9 vs. 22 ± 8, *p* < 0.01).

### 3.4 Responses to isometric handgrip exercise

In athletes, a pressor response occurred where, SBP and DBP increased during isometric handgrip exercise by 18 ± 11 and 18 ± 6 mm Hg, respectively. In athletes, average HR increased from 62 ± 8 bpm to 82 ± 11 bpm during exercise. The average The average maximum voluntary contraction in athletes was 39.2 ± 8.0 kg. The mean force of contraction was slightly above 30% of maximum voluntary contraction at 13.3 ± 3 kg for a total contraction time of 117.9 ± 56.7 s. The average rating of perceived exertion at the end of the exercise was 17 (range: 14–19). Three athletes (one women and two men) failed to increase their DBP greater than a 10 mmHg increase during handgrip.

### 3.5 Brachial artery flow-mediated dilation

Baseline brachial artery diameter and values of FMD, with and without adjustments for diameter, are shown in Table 2. As indicated in this table, average values are from 14 athletes and 10 control participants who were studied prior to the first confirmed case of COVID-19. Baseline diameter was greater in athletes compared to control participants (4.32 ± 0.63 vs. 3.54 ± 0.42 mm, *p* < 0.01). The FMD response was decreased in athletes when expressed as a percentage (6.50% ± 2.06% vs. 11.07% ± 4.09%, *p* < 0.01). However, FMD responses were similar between groups when allometrically scaled for baseline diameter (athletes: 10.0% ± 3.5% vs. 7.1% ± 2.4%, *p* = 0.058) and normalized by shear rate (athletes: 0.055% ± 0.026%/s-1, controls: 0.068% ± 0.049%/s-1, *p* = 0.40).

**TABLE 2:** Brachial artery flow-mediated dilation (FMD) expressed as a percentage change and normalized to shear AUC. Values of brachial artery diameter are presented at baseline and peak response for each variable. Two-tailed Student’s t tests for two samples of equal variance were performed between control (*n* = 7 M/3 F) and athletes with a SARS-CoV-2 infection (*n* = 10 M/4 F) groups. *p < 0.05, between groups. Allometrically corrected mean FMD were not different between groups. Data are mean ± SD.

	SARS-Cov-2-positive (athletes)	SARS-Cov-2-negative (FMD control)	
	*n* = 14 (10 men/4 women)	*n* = 10 (7 men/3 women)	*p*
Baseline diameter, mm	4.32 ± 0.63	3.54 ± 0.42*	< 0.01
Peak vasodilation diameter, mm	4.60 ± 0.66	3.92 ± 0.44*	< 0.01
Absolute change in diameter, mm	0.28 ± 0.07	0.39 ± 0.12*	0.01
FMD, %	6.50 ± 2.06	11.07 ± 4.09*	< 0.01
Allometrically scaled FMD, %	10.0 ± 3.5	7.1 ± 2.4	0.058
Shear rate, s^-1^	119.9 ± 31.5	177.7 ± 72.1*	0.01
FMD/shear, %/s^-1^ AUC	0.055 ± 0.026	0.068 ± 0.049	0.40

### 3.6 Responses to orthostatic challenge

**FIGURE 3:**
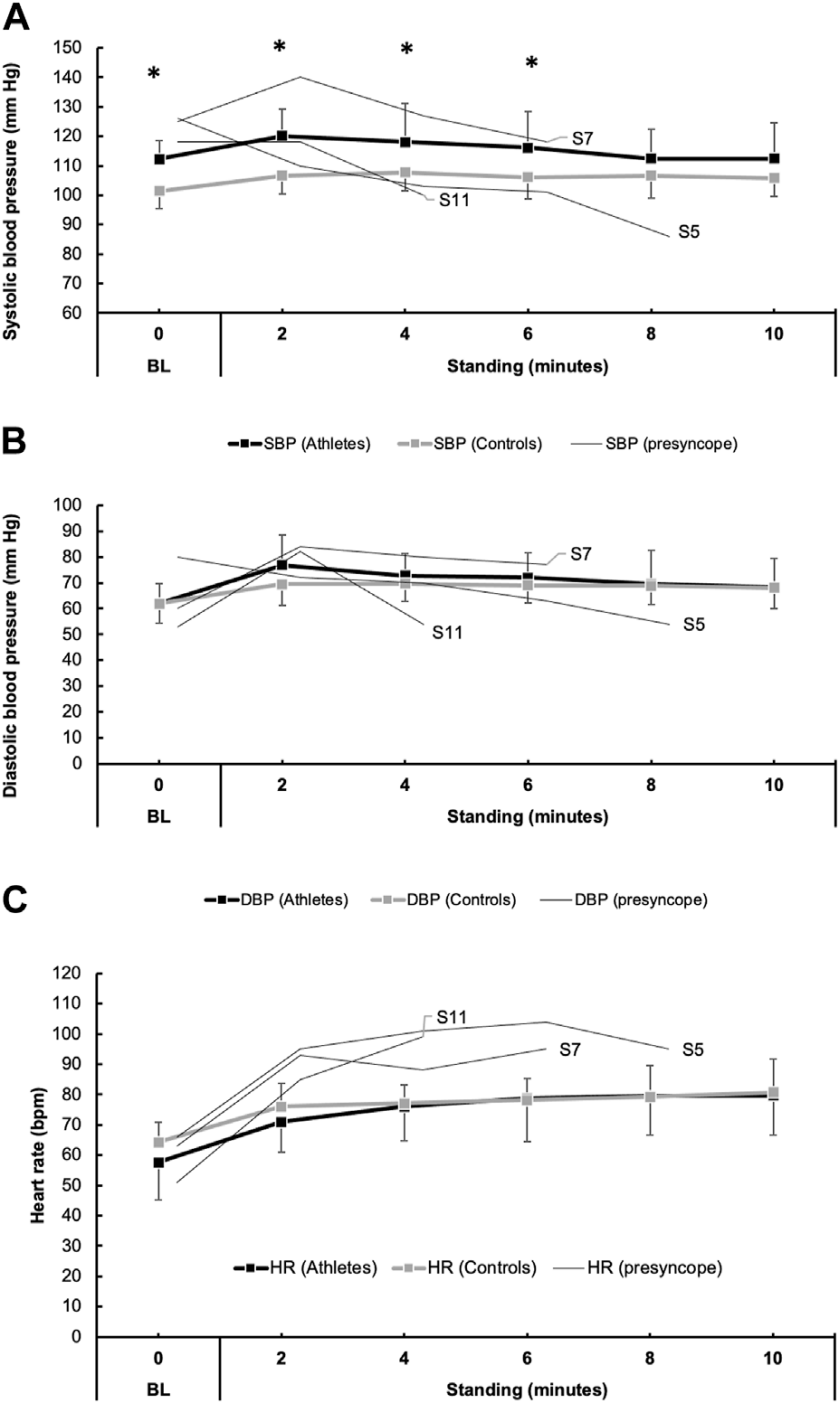
Blood pressure (BP) responses to orthostatic challenge in 16 athletes with COVID-19 and 16 individuals with no known COVID-19 diagnosis (controls). Panels **(A)** and **(B)** show the mean responses for systolic blood pressure (SBP, black boxes/lines) and diastolic blood pressure (DBP, gray boxes/lines). Additionally, thin lines represent the trend in BP in three athletes (participant number: 5, 7, and 11) who developed presyncopal symptoms and did not complete the 10-min orthostatic challenge. Similarly, heart rate (HR) responses are shown in **(C)**.

Individual and average SBP, DBP, and HR values during orthostatic challenge are shown in Figure 3. During the orthostatic challenge, three athletes became presyncopal and were placed in the seated position until symptoms improved. In the remaining 13 athletes that finished the protocol, when compared to 16 control participants, SBP was significantly elevated at baseline (*p* < 0.01) and during the first 6 minutes (*p* < 0.01) of standing (Figure 3). However, DBP during standing was similar between athletes and controls (Figure 3). HR increased similarly in both athletes (*p* < 0.01) and controls (*p* < 0.01) during the final minute of standing but was not different between groups (*p* = 0.82). When compared to a group of ten aerobically trained athletes (Aubert et al., 2001), during standing, athletes with COVID-19 had lower SDNN (64.0 ± 24.3 ms vs. 92.9 ± 30.9 ms, *p* < 0.05), RMSSD (30.0 ± 16.0 ms vs. 47.2 ± 11.1 ms, *p* < 0.05) and pNN50 (11.2% ± 12.3% vs. 22.4% ± 8.9%, *p* < 0.05).

### 3.7 Correlations to symptom scores

Athletes were mostly asymptomatic at the time of their study visit, except for six, who reported only lingering ageusia and anosmia. The mean length of symptoms was 4 ± 6 days, and the calculated symptom score was 4 ± 3 points. The list of reported symptoms included congestion or rhinorrhea (*n* = 8), fatigue (*n* = 6), new ageusia or anosmia (*n* = 6), cough (*n* = 5), fever or chills (*n* = 4), skeletal muscular or body aches (*n* = 4), headache (*n* = 3), sore throat (*n* = 2), and shortness of breath (*n* = 1).

In the athletes, symptom scores and responses to the VM were not correlated (Valsalva ratio: r = −0.01, *p* = 0.97, drop in MAP during phase IIE: r = −0.03, *p* = 0.93, and drop in MAP during IIL: r = 0.09, *p* = 0.79). Symptom scores and RSA amplitude, assessed during slow breathing, were not correlated (r = −0.28, *p* = 0.43. No relationships were observed between symptom scores and HRV indices of parasympathetic tone at rest (RMSSD: r = −0.29, *p* = 0.40, and HF: r = −0.29, *p* = 0.41) or during orthostatic challenge (RMSSD: r = 0 .06, *p* = 0.86, and HF: r = 0.08, *p* = 0.83). No relationships were observed between symptom scores and plasma renin activity (r = −0.26, *p* = 0.46) or aldosterone levels (r = 0.27, *p* = 0.44).

## 4 Discussion

The impact of a SARS-CoV-2 infection on cardiac autonomic function and vascular function in collegiate athletes was investigated. This study serves to expand the existing literature on the effects of COVID-19 and is the first report of autonomic and vascular function in collegiate athletes following a mild SARS-CoV-2 infection. Several atypical and borderline responses to common autonomic function tests were observed in 16 collegiate athletes, which may be suggestive of mild dysautonomia. When adjusted for shear rate, vascular function was not impaired in athletes when compared to control participants. These findings suggest that in the weeks following a positive SARS-CoV-2 infection, some athletes may show detectable alterations in autonomic function while systemic vascular function may remain largely unchanged.

Several reports have documented early signs of dysautonomia in young adults (Stute et al., 2021; Freire et al., 2022), as well as in older adults who developed severe COVID-19 infections requiring hospitalization (Freire et al., 2022). Interestingly, Freire et al. showed that autonomic impairments were worse in individuals who were physically inactive, overweight, or obese (Freire et al., 2022). It can be reasonably speculated that collegiate athletes, who have moderate to high levels of fitness secondary to competing at the Division I level, may have favorable outcomes compared to untrained individuals. Indeed, in one recent study, athletes with a higher peak VO2 were more likely to be asymptomatic (Vollrath et al., 2022). However, the cardiovascular risk as part of the post-acute sequelae of COVID-19 should not be overlooked in athletes (Goergen et al., 2021; de Abreu, 2022). Nonetheless, whether athletes have preserved autonomic function following COVID-19 has not been thoroughly investigated until now. While several atypical responses to autonomic function tests were observed in the present study, it is important to note that many of the responses to the battery of tests were within expected limits in some athletes. Much of this discussion will focus on these atypical findings.

Resting autonomic function in athletes with COVID-19. The parasympathetic nervous system typically dominates sympathetic activity at rest. However, during viral infections, increased circulating inflammatory cytokines are believed to drive this increase in sympathetic nerve activity, while the vagus nerve acts as a neuro-immuno-modulator pathway between the brain and peripheral inflammation. The indices of HRV have been used to assess sympathetic and parasympathetic function in young adults during the weeks following a SARS-CoV-2 infection (Stute et al., 2021; Freire et al., 2022; Skow et al., 2022). However, these previous reports found some conflicting differences in the HRV indices reflective of parasympathetic tone. Stute and colleagues reported an increase in parasympathetic tone while the most recent report by Skow and colleagues showed that HRV indices were unchanged compared to otherwise healthy individuals without a COVID-19 diagnosis and the parasympathetic and global variability improved in the time since the diagnosis (Stute et al., 2021; Skow et al., 2022). Consistent with the latter, in the present study, there were no appreciable differences in resting parasympathetic tone, assessed via HRV indices (e.g., RMSSD and HF power).

### 4.1 The responses to Valsalva maneuver in athletes with COVID-19

The VM is a tool used frequently by clinicians and physiology researchers to assess the cardiovascular system and neural circuits that innervate it. However, the use of the VM for autonomic function testing in individuals following a SARS-CoV-2 infection is limited to a few studies that utilized the maneuver as a part of a quantitative scored assessment. While the VM is commonly performed by athletes during exercise involving strenuous isometric contractions, BP and HR responses to VM are similar between physically trained and control participants (Fuenmayor et al., 1992; Looga, 2001). Thus, it is believed that in the present study, atypical responses to VM during phase II as seen in some athletes is not a normal phenomenon.

Figure 1 depicts a return map of individual MAP values, where the initial perturbation in MAP (IIE) is plotted along the *x*-axis, while the corrective influences of increased HR and peripheral resistance on MAP (IIL) are plotted along the *y*-axis. With each individual athlete’s phase IIE and IIL values arranged on this coordinate plane, it is quickly revealed that not all athletes have a reduction in MAP during phase IIE. Indeed, in some (4/16) athletes, MAP increased from baseline values during phase IIE, and remained elevated during the entirety of phase II. This response is commonly termed the “square-wave” or “flat top” response, which can be observed in patients with elevated left-sided filling pressure (Zema et al., 1980), as well as healthy individuals (Vogel et al., 2008). Effect of Position on Valsalva Maneuver. However, in otherwise healthy individuals, the occurrence of the square-wave pattern has been attributed to posture, where performing the VM in the supine posture increases the likelihood of observing this pattern (Vogel et al., 2008). However, in the remaining athletes whose BP was reduced during phase IIE, those with reductions greater than 20 mm Hg were considered mildly reduced in accordance with previously published criteria (Novak, 2011). This response was only observed in four athletes (participant number: 10, 11, 12, and 16) during one of the three VMs. During phase IIE, MAP values that did not return to or above values were considered abnormal per the previously published criteria (Novak, 2011). In these instances, it is believed that the baroreflex mediated increases in sympathetic tone, directed towards the heart and peripheral blood vessels, are insufficient in restoring MAP to or above baseline levels. Although, it is important to note that when taken in the context of the normative values (Novak, 2011), the athletes that displayed this perturbation in MAP could be classified only as mildly abnormal, as phase IIL MAP values did not fall below 20 mm Hg.

In Figure 1, the dashed line (*y* = *x*) represents a convergence of the phase IIE and IIL MAP values. In other words, an inflection point in MAP during phase II of the VM was not discernable. Individuals showing phase IIE reductions in MAP >20 mm Hg and an absence in phase IIL (i.e., convergence of the phase IIE and IIL) are considered moderately abnormal (Novak, 2011). However, in the present investigation, none of the athletes met this qualification. To assess the parasympathetic reactivity during recovery from the VM (i.e., phase IV), the ratio between the peak HR, which occurred during the VM, and the minimum HR in the 30 s preceding the maneuver is known as the Valsalva ratio. Previously defined normal values for healthy participants (ages: 10–29 years) are >1.59 in men and >1.46 in women (Ewing et al., 1985). In the present study, 10 of the 16 athletes were within normal limits. In agreement with resting HRV indices reflective of parasympathetic tone, it does not appear that athletes have changes in parasympathetic reactivity to the VM. However, in the individuals where BP did not return to baseline levels, it is suspected that this could be the result of impaired peripheral vasoconstriction as the phase IIE inflection point is typically indicative of sympathetic activation secondary to arterial baroreceptor unloading.

### 4.2 The respiratory sinus arrhythmia in athletes with COVID-19

The RSA test attempts to evoke a physiological phenomenon where cyclic oscillations in HR occur with slow and deliberate breathing. Increases and decreases in HR occur during the inspiratory and expiratory phases, respectively. This is caused by influences of the inspiratory centers on the cardioinhibitory centers located within the nucleus ambiguus, as well as reflex responses to oscillating BP and activation of pulmonary stretch receptors (Feher, 2017). According to previously defined normal values for healthy subject (ages: 10–29 years) (Novak, 2011), RSA amplitude (maximum-minimum HR) fell below the normal limits (≥14 bpm) in three athletes, which could be indicative of a mild alteration in parasympathetic function.

### 4.3 Isometric handgrip exercise in athletes with COVID-19

Isometric handgrip exercise is a physiological test used to elicit autonomic responses consisting of a decrease in parasympathetic tone and increases in sympathetic mediated pressor and cardioaccelerator response via increased central command and a reflex arc known as the exercise pressor reflex (Kaufman and Hayes, 2002). In the current investigation, BP and HR increased appropriately in response to a sustained isometric handgrip at 30% of maximal voluntary contraction in most athletes. This suggests that autonomic nervous system is not impaired in response to fatiguing isometric contraction. However, one athlete had a nearly 10-point reduction in SBP, and three athletes had DBP that failed to increase by > 10 mmHg, while all other athletes displayed pressor typical responses. Upon review the beat-by-beat BP tracing for the individual with reduced BP, no obvious artifact was appreciated and thus the tracing was not excluded from the analysis. Thus, it appears that there are minimal, if any, alterations in the pressor responses to isometric handgrip exercise or decrements in forearm grip strength in athletes with COVID-19. However, a comparison with a control group of COVID-negative athletes would provide a more complete picture.

### 4.4 Orthostatic intolerance observed in athletes with COVID-19

Among the findings with significant clinical relevance, three athletes developed orthostatic intolerance during 10 min of standing. These individuals met the recognized criteria for postural orthostatic tachycardia syndrome (POTS) based on their heart rate increase and documented BP change upon standing (Sheldon et al., 2015). Interestingly, it is important to note that since the start of the pandemic, a growing number of reports have documented an increase in individuals who now meet the criteria for disorders of orthostatic intolerance such as postural orthostatic tachycardia syndrome secondary to a COVID-19 diagnosis. The act of standing upright induces a gravitational shift in blood volume, causing blood to pool in the lower extremities’ highly distensible veins and thereby reducing cardiac preload. Normally, standing triggers an autonomic reflex that maintains BP through increased HR and peripheral vasoconstriction mediated by sympathetic activity. However, when the maintenance of BP upon standing is unsuccessful, symptoms of cerebral hypoperfusion may occur.

While exploring orthostatic intolerance in athletes with COVID-19, it is essential to recognize that previous investigations have reported both decreased and increased tolerance to orthostatic stress in endurance athletes (Levine et al., 1991a; 1991b; Convertino, 1993; Raven and Pawelczyk, 1993; Sugawara et al., 2012). This variability may be attributable to factors such as eccentric left ventricular hypertrophy commonly found in these athletes (Levine et al., 1991b). Exercise-associated postural hypotension is the most common example of this which accounts for nearly 60% of collapse at the conclusion of endurance and ultra-endurance events (Holtzhausen and Noakes, 1995). However, the present study aims to explore the possible correlation between COVID-19 and the observed orthostatic responses, rather than solely attributing the symptoms to athletic activity. Indeed, indices of global HRV (e.g., SDNN) and parasympathetic tone (e.g., RMSSD and pNN50) were lower in athletes with COVID-19 compared to ten athletes in a study conducted prior to the COVID-19 pandemic (Aubert et al., 2001). This would suggest that COVID-19 may contribute to a relative shift towards sympathetic dominance during standing in athletes. However, it is essential to recognize the value of comparing these findings with a control group of COVID-negative athletes. Unfortunately, due to constraints related to the COVID-19 pandemic, it was not feasible to collect these data. This limitation is duly noted in the limitations section below and is a substantial constraint of the present study.

### 4.5 Brachial artery flow-mediated dilation (FMD) and its implications in athletes with COVID-19

The FMD technique, leveraging Duplex ultrasound technology, measures the responses of conduit arteries to shear stress (Celermajer et al., 1992), providing crucial insights into vascular health. Previous investigations have indicated impaired endovascular function in both young adults (Ratchford et al., 2021) and trained athletes (Bauer et al., 2022) following SARS-CoV-2 infection. The virus binds to cells primarily via the angiotensin converting enzyme 2 (ACE2), a receptor found on various cell types, including those in the respiratory tract, heart, and blood vessels (Ferrario et al., 2005; Fehr and Perlman, 2015). Damage to the blood vessels and other parts of the circulatory system by SARS-CoV-2 may be caused by either direct infection secondary to ACE2 binding or via an inflammatory response characterized by cytokine production or other inflammatory mediators. In the present study, there was a 4.6% decrease in FMD in athletes with COVID-19. However, previous investigations in healthy adults reveal that FMD responses can be impacted by artery size (Thijssen et al., 2008) and exercise training can induce remodeling in conduit arteries, resulting in increases in diameter and reduced wall thickness (Green et al., 1996; Huonker et al., 2003; Rowley et al., 2012). Moreover, shear rate is inversely correlated with vessel diameter. Given the significant differences in baseline diameter, we conducted an allometric scaling analysis on the FMD data, as suggested by Atkinson and Batterham (Atkinson and Batterham, 2013). The allometrically scaled FMD data revealed no significant differences between the groups. Likewise, the FMD data, taking into account the area under the shear rate curve from deflation to maximal dilation, was not significantly different between the two groups.

### 4.6 Limitations

This study represents a balance between an experimental investigation and a case series, and several limitations should be acknowledged. A significant limitation was that the control groups included two cohorts, which did not undergo identical experimental measures or procedures and were not controlled for gender or physical activity levels. This inconsistency complicates the interpretation of results and points to the need for future research to control for these variables more comprehensively. The challenge in recruiting appropriate control participants was primarily due to institutional precautions taken in response to the COVID-19 pandemic, which had profound impacts on data collection and participant recruitment for approximately 2 years. Initially, student athletes participating in the study travelled exclusively from the university’s main campus to the College of Medicine as a part of their return-to-play protocols and, in addition, to participate in this research. Once these screening visits were relocated closer to the university’s main campus, it became unrealistic to recruit these athletes due to the travel time. Additionally, when the precautions and restrictions were lifted to study control athletes, it is probable that many potential athletic control participants would have contracted COVID-19, either with symptoms or asymptomatically. This reality further complicated the recruitment of suitable control groups. The control group for Flow-Mediated Dilation (FMD) measurements was obtained prior to the first known SARS-CoV-2 infection in the US, while the control group for the orthostatic tolerance test was collected between February and September 2022. Although these control participants did not have a positive COVID-19 diagnosis, the absence of confirmation of previous infection using viral titer is acknowledged as a limitation.

Another important limitation was the use of published normative values for several autonomic function tests. These values represent otherwise healthy individuals, but do not account for physical activity levels. At present, no established normative databases of sufficient size differentiate between physical activity levels for blood pressure (BP) or heart rate (HR) values in athletes for the autonomic function tests proposed by Novak et al. (Novak, 2011). The assumption was that collegiate athletes in the present study were highly physically active, given the demands of Division I level competition. However, due to pandemic precautions, we were unable to perform cardiopulmonary exercise stress testing on these individuals. Furthermore, national and statewide infection prevention strategies could have resulted in some athletes experiencing a decline in physical fitness due to event and practice cancellations. Consequently, it is difficult to determine whether variability in our findings is a result of varying degrees of physical fitness level among the athletes. Given the demonstrated prevalence of impaired autonomic and vascular function in young adults infected by SARS-CoV-2(Ratchford et al., 2021; Stute et al., 2021), documenting atypical findings in collegiate athletes is important. Future investigations, either appropriately controlled or aimed at establishing normative values in collegiate athletes for these autonomic tests, could help clarify the clinical significance of our findings.

Lastly, it is crucial to carefully interpret and communicate the implications of these findings. Although our results do highlight some unusual findings among athletes post-SARS-CoV-2 infection, these observations should not be immediately construed as clinically or diagnostically abnormal. Due to the numerous factors at play in such a complex environment as the human body under the stress of both elite athletics and viral infection, these results should be seen as preliminary. It is important to note that these findings, while intriguing, are exploratory in nature and do not warrant immediate changes in clinical practice or diagnostic criteria. They should rather serve as a basis for further, more controlled studies.

We must also guard against promoting the narrative that athletes or regular exercisers could be immune to SARS-CoV-2 infection and any associated adverse outcomes. The results of this study are intended to contribute to a growing body of knowledge and should be considered as one piece of a much larger puzzle. Thus, while we believe our findings add valuable insights to our understanding of SARS-CoV-2’s impact on athlete health, they must be interpreted with caution and within the broader context of ongoing research in this area.

### 4.7 Conclusion

These finding suggest that in the weeks following a mild acute SARS-CoV-2 infection, some collegiate athletes may show signs of autonomic impairment in the form of atypical HR and BP responses to a battery of tests designed to activate autonomic reflex pathways. However, systemic vascular function assessed via FMD, corrected for variations in baseline diameter and normalized by shear rate, does not appear to be impaired in athletes in the weeks after a mild acute SARS-CoV-2 infection. More appropriately controlled investigations are needed as the clinical significance of neural dysautonomia in this population could be important in understanding the post-COVID-19.

### 4.8 New findings and practical implications

This study examined hemodynamic responses to the Valsalva maneuver, slow breathing, fatiguing isometric handgrip exercise and orthostatic stress, as well as the flow-mediated dilation in athletes with COVID-19. Based on our findings, some collegiate athletes develop mild alterations in autonomic function, which is consistent with previous reports in young adults. In contrast, athletes did not exhibit altered the flow-mediated dilation when adjusted by baseline diameter and normalized by shear rate. This may have important implications for understanding the post-acute sequelae of COVID-19, such as dysregulation of the autonomic nervous system, in collegiate athletes.

## Data Availability

The original contributions presented in the study are included in the article/Supplementary material, further inquiries can be directed to the corresponding author.
